# Impact of caring for someone with a rare rheumatic condition, views from patients and informal carers—the need for cat-like vigilance

**DOI:** 10.1093/rap/rkz003

**Published:** 2019-02-01

**Authors:** Janice Mooney, Karly Graham, Richard A Watts

**Affiliations:** 1 School of Health and Social Care, University of Staffordshire, Stafford, UK; 2 Norwich Medical School, University of East Anglia, Norwich, UK

**Keywords:** ANCA-associated vasculitis, carers, challenges of a rare condition

## Abstract

**Objective:**

ANCA-associated vasculitis (AAV) is a rare multisystem disease. Modern therapeutic protocols have turned AAV from an acute, frequently fatal disease into a chronic disease requiring long-term immunosuppression. Patients must often manage substantial burdens related to chronic illness and treatment-related side effects, requiring help from informal carers. This study aimed to explore the experience of patients and of informal carers of patients with AAV about the impact of managing a rare rheumatic condition.

**Methods:**

A qualitative approach using semi-structured interviews was used. Interviews were conducted with a purposeful sample of 18 pairs of patients with AAV and their informal carers. The interviews were used to explore the participants’ experience and effects of caring. The interviews were recorded and transcribed as verbatim text and analysed using the framework technique.

**Results:**

Eighteen patients (seven female; disease: 10 granulomatosis with polyangiitis; four microscopic polyangiitis; four eosinophilic granulomatosis with polyangiitis; age range 34–78 years; disease duration 1–20 years). Caregiver and patient perspectives were shared. The emerging themes were the physical and psychological impacts of the disease, the need for constant vigilance and fear of the future.

**Conclusion:**

Both patients and carers faced a range of challenges in managing a rare condition, including the seriousness of the illness, dealing with the emotional toll and knowing what to expect. This study offers insight into the experiences of patients and informal carers, and health-care professionals should address individuals’ fears and expectations for recovery.

**Key messages**

- Carers play a key role in the management of patients with ANCA-associated vasculitis.
- Carers carry a heavy burden of responsibility when caring for someone with ANCA-associated vasculitis.
- Carers require support in their own right from health-care professionals.

## Introduction

The ANCA-associated vasculitides (AAVs) granulomatosis with polyangiitis (Wegener’s), eosinophilic granulomatosis with polyangiitis (Churg Strauss) and microscopic polyangiitis are a group of rare, potentially life-threatening conditions, which can be fatal if untreated. Many organs can be affected, such as the kidney, heart, lung, upper and lower airways and the nervous system. Modern immunosuppressive therapy has changed the outlook for patients with AAV from being very poor with a high mortality to a chronic disease associated with a need for long-term treatment [1]. Despite improvements in survival, there are significant side effects of therapy, such as increased risk of infection, hypertension, osteoporosis, diabetes associated with CSs and haematological and skin malignancies [2–7]. 

The diagnosis of AAV has a physical, psychosocial and financial impact on patients’ lives [8–12]. The physical impact can cause a range of problems, including hearing loss, blindness, shortness of breath, saddle nose deformity and nerve damage [3, 13]. Disease-related complications, such as stroke, myocardial infarction, kidney failure, cancer, blindness, stomach ulcer and seizures, have been reported in 10% of patients [14–16]. At 5 years, the long-term side effects of CSs are substantial: 41% had hypertension, 38% osteoporosis, 28% diabetes mellitus and 25% had developed cataracts [3]. Fatigue is a major cause of impaired physical health and appears to be unresponsive to treatment [17, 18]. Patients must often manage substantial burdens related to chronic illness and treatment-related side effects [3, 8, 10]. It is clear that the impact of a diagnosis of AAV is significant, and patients need help and support to manage their disease.

Informal carers are people who actively participate in the care of a patient on a practical and/or emotional basis, usually partners or family members. To date, there is no knowledge about the role played by informal carers in the treatment and lives of patients with AAV, and we need a better understanding of their role.

The aim of this study was to explore the experience of patients and informal carers of patients with AAV about the impact of managing a rare rheumatic condition.

## Methods

### Design

A qualitative approach using semi-structured interviews was used because it enables the researcher to seek the views and opinions of individuals and gain an understanding and insight of their social world [19]. Semi-structured, one-to-one interviews allow the researcher to explore topics in depth, discovering rich experiential data from participants about their experiences. It allows the researcher to follow up interesting responses and adapt questions, which is not possible with self-administered questionnaires [20]. An interview guide was prepared with a list of questions to facilitate this (supplementary data, available at *Rheumatology Advances in Practice* online), which was informed by the existing literature, patients and three health-care professionals (one nurse, one consultant and one qualitative researcher) to agree the topics for discussion. This was piloted with three patients and three carers.

### Participants

A purposeful sample of 18 pairs of patients with AAV and their informal carers were recruited in East Anglia (Tables 1 and 2). Patient and carer pairs were enrolled from two vasculitis specialist centres, with the consultant or vasculitis specialist nurse approaching potential participants, providing an information sheet and consent forms. If both patient and carer agreed to participate, they posted the consent form to the researchers, who contacted them directly and arranged the interview. Consent was obtained separately from patient and carer, with the patient being asked to consent to their carer sharing personal information about them.

**Table 1:** Patient characteristics

Participant	Gender	Age (years)	Disease duration (years)	Disease
P1	M	68	6	GPA
P2	F	34	2	EGPA
P3	M	55	2	GPA
P4	M	76	7	MPA
P5	M	74	7	MPA
P6	M	71	7	EGPA
P7	M	76	3	EGPA
P8	F	68	16	EGPA
P9	F	72	1	MPA
P10	F	74	20	GPA
P11	M	66	3	GPA
P12	M	70	7	GPA
P13	F	55	2	GPA
P14	M	76	14	GPA
P15	M	60	14	GPA
P16	F	76	15	GPA
P17	M	68	7	GPA
P18	F	67	5	MPA

EGPA: eosinophilic granulomatosis with polyangiitis; F: female; GPA: granulomatosis with polyangiitis; M: male; MPA: microscopic polyangiitis.

**Table 2:** Carer characteristics

Participant	Gender	Age (years)	Employment status	Relationship
C1	F	66	Retired	Partner
C2	M	38	Full time	Partner
C3	F	42	Full time	Partner
C4	F	78	Retired	Partner
C5	F	70	Retired	Partner
C6	F	71	Retired	Partner
C7	M	76	Retired	Partner
C8	M	70	Retired	Partner
C9	M	75	Retired	Partner
C10	M	70	Retired	Partner
C11	M	66	Retired	Partner
C12	F	66	Retired	Partner
C13	M	65	Part time	Partner
C14	F	45	Full time	Partner
C15	M	60	Retired	Partner
C16	F	76	Retired	Partner
C17	M	68	Retired	Partner
C18	F	67	Retired	Partner

F: female; M: male.

Patients and carers were interviewed separately in order to obtain their independent views on the role of carer, because these issues were often not discussed with each other. Interviews took place either in the hospital or in the participants’ home, or at a convenient location. Interviews lasted between 45 min and 1 h. Ethical approval for the study was provided by the Norfolk Research Ethics Committee (ref. 16/EM/0190).

### Inclusion and exclusion criteria

Participants (both carers and patients) were aged >18 years, with an adequate command of English. Patients had a confirmed diagnosis of AAV, classified using the European Medicines Agency approach [21]. All had to be capable of giving informed consent. Informed consent was obtained. Patients and carers with severe medical conditions were not recruited. 

### Data analysis

The interviews were recorded on a digital recorder and transcribed as verbatim text. The transcriptions were read and analysed by two authors (J.M., K.G.) using the framework technique [22], a five-step process that involves: familiarization with the data; identification of a thematic framework; indexing; charting; and mapping and interpretation.

This technique is a systematic and comprehensive method for researchers to analyse and make sense of data by mapping emergent themes or concepts that explain those data. Data were coded into developing descriptive categories; these were recorded onto charts, then analysed for patterns and mapped into key themes. This framework was then applied to the other transcripts, and the themes were developed further. The framework headings and subheadings identified were member checked: (a) a summary of headings identified was sent to interviewees; and (b) they were invited to discuss their views on their clarity and authenticity in a telephone conversation with the facilitator to ensure the interpretation of the data was an accurate representation and understanding of participants’ views. Participants agreed with the themes and subheadings; no new categories emerged, and no existing ones were amalgamated.

## Results

**Fig. 1:**
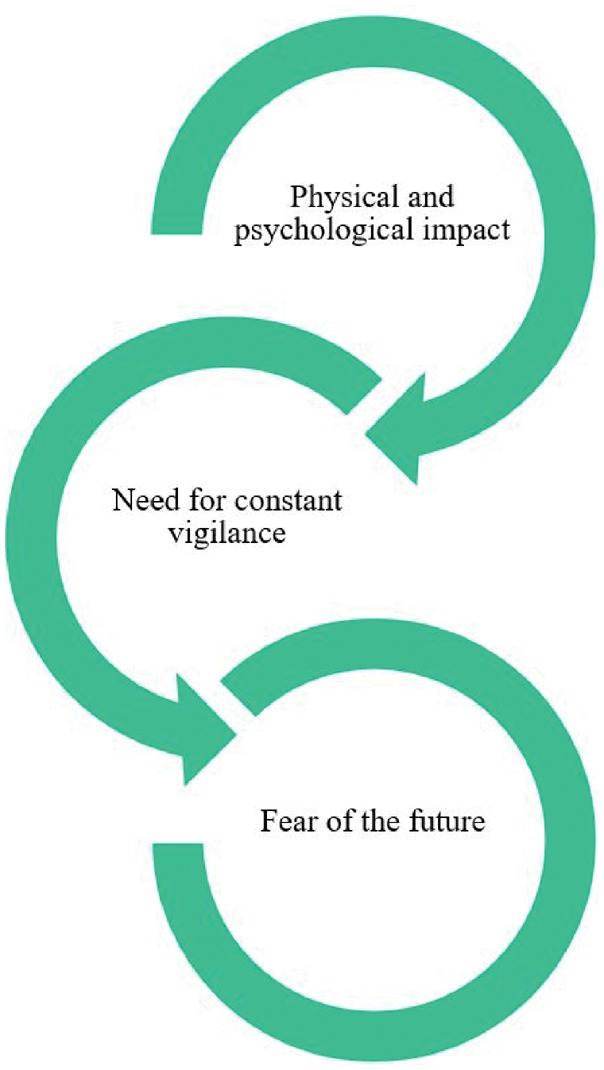
Emergent themes

There were 18 patients (seven female; disease: 10 granulomatosis with polyangiitis; four microscopic polyangiitis; four eosinophilic granulomatosis with polyangiitis; age range 34–78 years; disease duration 1–20 years). Details are provided in Tables 1 and 2. Caregiver and patient perspectives were shared. The emerging themes were the physical and psychological impacts of the disease, the need for constant vigilance and fear of the future (Fig. 1).

### Physical and psychological impact of the disease

This theme describes the physical and emotional impact of the disease, particularly during the acute phase of the illness. Patients reported extreme tiredness and weakness, being unable to walk and needing help with dressing and bathing. P3: “Yes, I fall asleep anywhere. And the fatigue—people don’t believe you’re fatigued, they just think you’re lazy.” P3: “It’s worse for her than it is for me, because I’m ill. She’s getting all this and she ain’t ill, bless her. She’s missing out on so much. It’s hard. There’s no support for her whatsoever. She’s had to dress me some days.” P9: “When I was really ill, he was actually doing everything.”

Carers told of the physical help they provided and the psychological and emotional toll of caring for someone with a serious rare illness. C5: “When he came home—finally—it was the most frightening time, because he was wasted away. He’d lost so much weight, like a skeleton. And just walking to make a cup of tea was too much. It took months of work to bring back his strength.” C10: “Am I selfish? I get a little bit cross when she gets tired, but I also get cross with her when she brings it on—between the last two operations, she’s out there digging holes in the garden! And puffing. We do snap at each other more than we used to. I probably don’t know how she feels.”

Both patients and carers described the depression and mood changes associated with CSs. P2: “It was really difficult, especially when I was on high-dose steroids—I was horrible, like a monster. He did say to me I was horrible. I think he used harsher words than that.” C13: “At that time, he was on very high doses of steroids and that just amplified all the emotions. He would fly off the handle at ridiculous things. I’d be thinking, ‘You’ve been half dead and you were alright with that, but the fact that the last Kitkat’s been eaten?!’.” Patients and carers also reported the emotional impact of the illness. C8: “It would be nice to talk to somebody who cares for someone in the same position. That would be nice—to know that you’re not on your own. I need to vent. Nobody understands. We’ve got lots of friends, but nobody understands. I look normal, but after 10 min work I’m exhausted. I’ve put in a bath lift upstairs. That does help me because I used to have to lift her in and out.” C5: “He does get depressed sometimes, but I get depressed sometimes too; I’ve got nobody to buck me up—do you know what I mean?”

Carers described the impact of having to take time off work and trying to work around this. C1: “My boss was brilliant—come in when you can. When it’s over you can work extra hard.” C2: “My shifts changed at work, so I could do the school run, go to work and come back to pick them up. I didn’t do anywhere near 40 h. So I did more childcare.” C4: “Now he’s got that money [carers’ allowance], I’ve got someone coming in to clean the oven.” C3: “Changing my work times round—my bosses were absolutely brilliant, by the way. I’ve had to take a drop in money now and I’m no longer a manager, just a [lower position], so it takes the strain off of him if I’m at home. Yes, and if I’d have had a worse boss, that would have directly cost me my job because I have to take so much time off.”

### Need for constant vigilance

This theme describes the constant background monitoring that carers and patients articulated. P8: “I had a bad infection and ended up with sepsis. I don’t now take anything for granted. She is always looking out for me. She used to be the one to call the ambulance, now if I felt that way, I’d go to A&E.”

P7: “He does worry—I’d think twice before I said anything because he would act immediately. So he would possibly panic a little bit. On the whole, considering he was never a medical person, he’s learned very well. I can’t complain if someone’s watching over me, can I? If there’s something I’m not realizing is happening to me, he would probably have noticed. He’s really vigilant and doesn’t particularly like leaving me alone, so yes, it must have had an impact.” In the context of a potentially serious illness, ‘keeping an eye on’ meant permanent vigilance for signs that the disease might be returning.

C1: “When he was first diagnosed, we did not go to bed. He sat in the chair. He was worried he’d got these nose bleeds. I honestly thought he was going to bed and would die. He slept on the settee down here and I put two chairs together and we did that every night. He wasn’t willing to go to bed because his mind was active, I wasn’t prepared to go to bed and leave him down there how he was feeling.” Others described how they checked on their partner’s breathing as a way of monitoring their condition. C10: “But the first thing I do every morning is listen for breathing. I don’t know if she knows that—I might have told her. Sometimes she’s very quiet—mostly you can hear [indicates laboured breathing], but sometimes I feel across to see if there’s a warm hand. I feel guilty that I don’t know what the illness is all about.”

C1: “Just, just keeping an eye on. One of the most frightening things before he got his diagnosis was his breathing at night. It wasn’t just snoring—it sounded as though he was drowning. I was kicking him to wake him up and sometimes he wouldn’t wake up. So when he started on his chemotherapy, it worked very quickly. I still wake up at night and check his breathing.”

### Fear for the future

This theme describes the fear that individuals experienced, particularly when they did not know what to expect. For those patients admitted to intensive care, their partners described emotional turmoil, especially dealing with the uncertainty of whether or not their partner would survive this life-threatening illness. C13: “And that afternoon he was blue-lighted up to hospital with me driving behind. Then another month in hospital, then deep vein thrombosis to add to it! I remember that first weekend in ITU, acute respiratory care, and sitting in the waiting room at 1 am, so lonely, waiting to hear. And on the walls was all this bereavement advice!” C15: “She was in hospital for about 5 weeks, 3 weeks of it in intensive care. I was told not to move too far from the telephone. It was quite an experience. There is that concern that we’re both getting older. I am aware that the vasculitis will shorten her lifespan.” The severity of the illness was often a shock. C2: “My worry is that I know one day we’ll go to bed and I’ll wake up and she won’t. It could be tomorrow.” P1: “It’s always in the back of your mind. Until I got this shock. I never used to worry about nothing—absolutely nothing. If the house fell down I didn’t really care. But since I got this—corr.”

Both patients and carers described triggers of emotional distress about what the future held and the inability to plan ahead. P1: “Well, we ain’t been on holiday for years. She’d like to go but I said I daren’t get on a plane, what with blood clots. And apart from that, if you’re on a plane and you’ve got all them germs going about. I used to love going on holiday. I say to her—next year we’ll go on holiday in this country. But at the time I just couldn’t be bothered. I felt so ill, stopped going on holiday.”

Patients also worried about the future and their life expectancy, with many contemplating what might happen. P5: “I do wonder what’s going to happen. Because the kidney could fail again and I’d have to go through all that again. And at my age they might not give me one so quick.”

P9: “I think I’m going to be fine, but I do think about things now (death) I do—where I’m going with this.”

P3: “I also worry about long-term health, life expectancy. Is this going to affect my life expectancy?”

## Discussion

In this study we have, for the first time, explored the role of carers in the lives of patients with a rare chronic rheumatic disease with a potentially severe outcome. To our knowledge, this has never been studied before. The three emergent themes were the physical and psychological impact of the disease, the need for constant vigilance and fear of the future (Fig. 1). All carers described the physical and emotional toll of caring, similar to that seen in carers of parents with OA [23], in which three themes emerged: the physical and emotional work of caring, changes in family roles and the inequity of caring within the family. A study of family caregivers for patients with RA/AS found that the primary consequences were loss of purchasing power, work problems, social isolation and emotional stress [24]. All the carers in our study experienced high stress levels relating to their partner’s diagnosis, a finding also seen in the cancer literature and in people with a rare genetic neurological disease (idiopathic basal ganglia calcification) [25–28].

Our study found that at diagnosis all patients described the psychological impact and physical impairments, a similar finding to others [8, 19, 29–31]. A study of 692 vasculitis patients found that they believed that their condition had affected their functional ability and emotional wellbeing [32]. A study of 410 AVV patients found that 74.8% reported high levels of fatigue associated with several factors, of which disturbed sleep and pain were the most important [33]. Participants in our study told of problems dealing with fatigue and sleepless nights. The diagnosis of a rare rheumatic condition impacts both the patient and the carer, especially during the acute phase of the illness, when patients can be critically ill. A literature review of the experiences of relatives of intensive care unit patients found they suffered anxiety, depression and fear [34].

After the acute phase of the illness, patients require regular and careful monitoring [2, 35]. Our study found that ‘keeping an eye’ on the person made up the majority of the care provided, a similar finding to other studies of carers [36]. This constant monitoring is mirrored in the cancer literature, because fear of recurrence is high in carers and cancer survivors [37]. Fear of disease progression is seen in patients with RA, diabetes and SSc [38, 39].

In our study, carers found this constant monitoring draining, because they were concerned about the potentially serious consequences for the patient if they did not act appropriately. This was one of the main drivers for ‘fear of the future’; neither carer or patient knew clearly what would happen next or what to look out for, feeling as if they were on a knife-edge between wellness (i.e. stable, controlled disease requiring little if any immunosuppression) and serious illness with uncontrolled vasculitis. Relapse is unpredictable, with at least half of patients with AAV experiencing a relapse at 5 years [40]. However, for patients and carers the symptoms of a relapse are not easily recognized, making self-management difficult. Indeed, expert clinicians find it a challenge, because no single test can predict relapse. A study comparing physician and patient global assessment scores of disease activity in 180 granulomatosis with polyangiitis patients found disparity in the results [41], with patients able to detect a rise in disease activity 3 months before their physicians. Therefore, it is important that patients’ perceptions of their disease activity are taken into account when assessing them [42]. There are several challenges for patients and carers in managing a rare condition: little or no previous experience and knowledge to draw upon, dealing with an unpredictable condition, the risk of relapse and the seriousness of treatment toxicity and side effects.

Many recognized the side effects of CSs, describing changes in personality, of anger and aggression attributed to high-dose CSs. This is a similar finding to AAV patients’ perceptions of the use of CSs, who knew their positive benefits but were concerned about side effects [9]. They also described a range of emotional factors related to CSs, including anger, anxiety, depression and mood swings.

A systematic review on the challenges of living with a rare condition found that there were substantial psychological, physical, social and emotional impacts [43]. These opinions are consistent with the present study. Fear of the future and dealing with uncertainty is common in the rare disease literature [44, 45]. Patients with chronic conditions often become experts on their own condition, but this is challenging in rare conditions. Patients with AAV reported difficulty adhering to complex medication regimes and understanding when to take their medication, and were slow to report symptoms and medication side effects [46]. They believed that medication side effects would go away and did not want to trouble their doctor about these. This could be attributed to a lack of patient education, because we have shown previously that informational needs about medication are high in this group [47]. It could be that information was either not provided or given at an inappropriate time, because patients have difficulty assimilating detailed information when acutely ill [8]. In our study, some carers reminded patients when to take their medication, and this is known to be associated with improved medication adherence [48]. We also found that as patients and carers became more experienced at living with AAV, they felt increasingly empowered to make decisions on the severity of the illness and act upon it.

### Strengths

The study has a number of strengths. We interviewed each pair of patients and carers separately on the same day. This helps to maintain independence of the interviews and thus obtain the distinct views of carers and patients.

### Weaknesses

This study has several potential weaknesses. We could not validate the type of care and time spent in caring. There may be recall bias for the interviews, and both patient and carer may have under- or overestimated the level of support needed/provided. However, this was minimized by interviewing both patient and carer on the same day.

### Conclusion

This is the first study of the role that informal carers provide to patients with AAV, a rare chronic rheumatic disease, and it makes a significant contribution to our knowledge. The study suggests that the role of carers is under-recognized; in particular, the emotional toll. All the carers in our study experienced high stress levels relating to their partner’s diagnosis, and the need for constant vigilance was draining. Health-care professionals should address the individual’s fears and expectations and ask carers how they are coping and if they require any support.

## Supplementary Material

Supplementary Data

## References

[1] Flossmann O, Berden A, de Groot K et al Long-term patient survival in ANCA-associated vasculitis. Ann Rheum Dis 2011;70:488–94. 21109517 10.1136/ard.2010.137778

[2] Robson JC, Dawson J, Cronholm PF et al Health-related quality of life in ANCA-associated vasculitis and item generation for a disease-specific patient-reported outcome measure. Patient Relat Outcome Meas 2018;9:17–34. 29379322 10.2147/PROM.S144992 PMC5759851

[3] Robson J, Doll H, Suppiah R et al Damage in the ANCA-associated vasculitides: long-term data from the European Vasculitis Study Group (EUVAS) therapeutic trials. Ann Rheum Dis 2015;74:177–84. 24243925 10.1136/annrheumdis-2013-203927

[4] Mahr A, Heijl C, Le Guenno G., Faurschou M. ANCA-associated vasculitis and malignancy: current evidence for cause and consequence relationships. Best Pract Res Clin Rheumatol 2013;27:45–56. 23507056 10.1016/j.berh.2012.12.003

[5] Bhamra K, Luqmani R. Damage assessment in ANCA-associated vasculitis. Curr Rheumatol Rep 2012;14:494–500. 22983618 10.1007/s11926-012-0291-1

[6] Phillip R, Luqmani R. Mortality in systemic vasculitis: a systematic review. Clin Exp Rheumatol 2008;5:94–104. 19026150

[7] Knight A, Askling J, Granath F, Sparen P, Ekbom A. Urinary bladder cancer in Wegener’s granulomatosis: risks and relation to cyclophosphamide. Ann Rheum Dis 2004;63:1307–11 Epub2004/05/08 15130900 10.1136/ard.2003.019125 PMC1754772

[8] Mooney J, Poland F, Spalding N, Scott DGI, Watts RA. In one ear and out the other—it’s a lot to take in: a qualitative study exploring the informational needs of patients with ANCA associated vasculitis. Musculoskeletal Care 2013;11:51–9. 22778039 10.1002/msc.1030

[9] Robson JC, Dawson J, Cronholm PF et al Patient perceptions of glucocorticoids in anti-neutrophil cytoplasmic antibody-associated vasculitis. Rheumatol Int 2018;38:675–82. 29124398 10.1007/s00296-017-3855-6 PMC5854718

[10] Carpenter D, DeVellis RF. (2011) Quality of life issues in vasculitis, advances in the etiology, pathogenesis and pathology of vasculitis. Amezcua-Guerra L (Ed.). http://www.intechopen.com/books/advances-in-the-etiology-pathogenesis-and pathology-of-vasculitis/quality-of-life-issues-in-vasculitis (10 October 2018, date last accessed).

[11] Newall C, Schinke S, Savage CO, Hill S, Harper L. Impairment of lung function, health status and functional capacity in patients with ANCA-associated vasculitis. Rheumatology 2005;44:623–8. 15695298 10.1093/rheumatology/keh548

[12] Cotch MF. The socioeconomic impact of vasculitis. Curr Opin Rheum 2000;12:20–3. 10.1097/00002281-200001000-00004 10647950

[13] Walsh M, Mukhtyar C, Mahr A et al Health-related quality of life in patients with newly diagnosed antineutrophil cytoplasmic antibody-associated vasculitis. Arthritis Care Res 2011;63:1055–61. 10.1002/acr.20471 PMC3128179 21452254

[14] Herlyn K, Hellmich B, Seo P, Merkel PA et al Patient-reported outcome assessment in vasculitis may provide important data and a unique perspective. Arthritis Care Res 2010;62:1639–45. 10.1002/acr.20276 PMC3123033 20556814

[15] Seo P, Min Y-I, Holbrook JT et al Damage caused by Wegener’s granulomatosis and its treatment: prospective data from the Wegener’s Granulomatosis Etanercept Trial (WGET). Arthritis Rheum 2005;52:2168–78. 15986348 10.1002/art.21117

[16] King C, Harper L. Avoidance of harm from treatment for ANCA-associated vasculitis. Curr Treatm Opt Rheumatol 2017;3:230–43. 29201630 10.1007/s40674-017-0082-y PMC5694500

[17] McClean A, Morgan MD, Basu N et al Physical fatigue, fitness, and muscle function in patients with antineutrophil cytoplasmic antibody-associated vasculitis. Arthritis Care Res 2016;68:1332–9. 10.1002/acr.22827 26713864

[18] Basu N, Jones GT, Fluck N et al Fatigue: a principal contributor to impaired quality of life in ANCA-associated vasculitis. Rheumatology 2010;49:1383–90. 20400759 10.1093/rheumatology/keq098 PMC3091420

[19] Parahoo K. Nursing research: principles, process and issues, 2nd edn. Basingstoke: Palgrave Macmillan, 2006.

[20] Robson C. Real world research, 2nd edn. Oxford: Blackwell, 2002.

[21] Watts R, Lane S, Hanslik T et al Development and validation of a consensus methodology for the classification of the ANCA-associated vasculitides and polyarteritis nodosa for epidemiological studies. Ann Rheum Dis 2007;66:222–7. 16901958 10.1136/ard.2006.054593 PMC1798520

[22] Ritchie J, Spencer L. Qualitative data analysis for applied policy research In: Bryman A, Burgess RG, eds. Analysing qualitative data. London: Routledge, 1994.

[23] Barker KL, Minns Lowe CJ, Toye F. ‘It is a big thing’: exploring the impact of osteoarthritis from the perspective of adults caring for parents – the sandwich generation. Musculoskeletal Care 2016;15:49–58. 27074876 10.1002/msc.1139

[24] Alfaro N, Lázaro P, Gabriele G et al Perceptions, attitudes and experiences of family caregivers of patients with musculoskeletal diseases: a qualitative approach. Rheumatol Clin 2013;9:334–9. 10.1016/j.reuma.2013.04.014 23871505

[25] Sherwood PR, Given BA, Doorenbos AZ, Given CW. Forgotten voices: lessons from bereaved caregivers of persons with a brain tumour. Int J Palliat Nurs 2004;10:67–75. 15039610 10.12968/ijpn.2004.10.2.12460

[26] Schmer C, Ward-Smith P, Latham S, Salacz M. When a family member has a malignant brain tumour: the caregiver perspective. J Neurosci Nurs 2008;40:78–84. 18481737 10.1097/01376517-200804000-00006

[27] Braun M, Mikulincer M, Rydall A, Walsh A, Rodin G. Hidden morbidity in cancer: spouse caregivers. J Clin Oncol 2007;25:4829–34. 17947732 10.1200/JCO.2006.10.0909

[28] Kim Y, Schulz R. Family caregivers’ strains: comparative analysis of cancer caregiving with dementia, diabetes, and frail elderly caregiving. J Aging Health 2008;20:483–503. 18420838 10.1177/0898264308317533

[29] Takeuchi T, Muraoka K, Yamada M, Nishio Y, Hozumi I. Living with idiopathic basal ganglia calcification 3: a qualitative study describing the lives and illness of people diagnosed with a rare neurological disease. SpringerPlus 2016;5:1713. 27777849 10.1186/s40064-016-3390-z PMC5050183

[30] Walsh M, Merkel PA, Mahr A et al The effects of glucocorticoid therapy on relapse rate in antineutrophil cytoplasm antibody-associated vasculitis: a meta-analyses. Arthritis Care Res 2010;62:1166–73. 10.1002/acr.20176 PMC2946200 20235186

[31] Koutantji M, Harrold E, Lane SE et al Investigation of quality of life, mood, pain, disability, and disease status in primary systemic vasculitis. Arthritis Rheum 2003;49:826–37. 14673970 10.1002/art.11471

[32] Grayson PC, Amudala NA, Mcalear CA et al Illness perceptions and fatigue in systemic vasculitis. Arthritis Care Res 2013;65:1835–43. 10.1002/acr.22069 PMC3962511 23861259

[33] Basu N, McClean A, Harper L et al Explaining fatigue in ANCA-associated vasculitis. Rheumatology 2013;52:1680–5. 23740186 10.1093/rheumatology/ket191

[34] McAdam JL, Puntillo K. Symptoms experienced by family members of patients in intensive care units. Am J Crit Care 2009;18:200–9; quiz 210. 19411580 10.4037/ajcc2009252

[35] Yates M, Watts RA, Bajema IM et al EULAR/ERA-EDTA recommendations for the management of ANCA-associated vasculitis. Ann Rheum Dis 2016;75:1583–94. 27338776 10.1136/annrheumdis-2016-209133

[36] Beesley L. Informal care in England: background paper to the wanless social care review. London: The Kings Fund, 2006.

[37] Simard S, Thewes B, Humphris G et al Fear of cancer recurrence in adult cancer survivors: a systematic review of quantitative studies. J Cancer Surviv 2013;7:300–22. 23475398 10.1007/s11764-013-0272-z

[38] Dankert A, Duran G, Engst-Hastreiter U et al Fear of progression in patients with cancer, diabetes mellitus and chronic arthritis. [In German.] Rehabilitation 2003;42:155–63. 12813652 10.1055/s-2003-40094

[39] Kwakkenbos L, Delisle VC, Fox RS et al Psychosocial aspects of scleroderma. Rheum Dis Clin North Am 2015;41:519–28. 26210133 10.1016/j.rdc.2015.04.010

[40] Smith RM, Jones RB, Guerry MJ et al Rituximab for remission maintenance in relapsing antineutrophil cytoplasmic antibody-associated vasculitis. Arthritis Rheum 2012;64:3760–9. 22729997 10.1002/art.34583

[41] Tomasson G, Davis JC, Hoffman GS et al Brief report: The value of a patient global assessment of disease activity in granulomatosis with polyangiitis (Wegener’s). Arthritis Rheum 2014;66:428–32. 10.1002/art.38248 24504815

[42] Specks U. Accurate relapse prediction in ANCA-associated vasculitis—the search for the holy grail. J Am Soc Nephrol 2015;26:505–7. 25324503 10.1681/ASN.2014080817 PMC4341490

[43] von der Lippe C, Diesen PS, Feragen KB. Living with a rare disorder: a systematic review of the qualitative literature. Mol Genet Genomic Med 2017;5:758–73. 29178638 10.1002/mgg3.315 PMC5702559

[44] Dures E, Morris M, Gleeson K, Rumsey N. The psychosocial impact of epidermolysis bullosa. Qual Health Res 2011;21:771–82. 21343430 10.1177/1049732311400431

[45] Schouffoer AA, Zirkzee EJ, Henquet SM et al Needs and preferences regarding health care delivery as perceived by patients with systemic sclerosis. Clin Rheumatol 2011;30:815–24. 21243390 10.1007/s10067-010-1645-6 PMC3101347

[46] Thorpe CT, DeVellis RF, Blalock SJ et al Patient perceptions about illness self-management in ANCA-associated small vessel vasculitis. Rheumatology 2008;47:881–6. 18403403 10.1093/rheumatology/ken126 PMC4084613

[47] Mooney J, Spalding N, Poland F et al The informational needs of patients with ANCA-associated vasculitis—development of an informational needs questionnaire. Rheumatology 2014;53:1414–21. 24625507 10.1093/rheumatology/keu026 PMC4103516

[48] Pepper J, Carpenter D, DeVellis R. Does adherence-related support from physicians and partners predict medication adherence for vasculitis patients? J Behav Med 2012;35:115–23. 22350097 10.1007/s10865-012-9405-5

