# Supplementary material for: Impact of caring for someone with a rare rheumatic condition, views from patients and informal carers—the need for cat-like vigilance
Source: Rheumatol Adv Pract. 2019 Feb 1;3(1):rkz003. doi: 10.1093/rap/rkz003 (PMC6649977; doi:10.1093/rap/rkz003)
Supplement: Supplementary Data [file rkz003_supp.doc]

# SUPPLEMENTARY MATERIAL

# Semi –Structured Interview Guide for Face to face interviews

**Facilitator Questions** **- Carers**

1) When you become a carer for a person with vasculitis, what informational needs did you have?

2) Have you been given any specific information/ leaflets specifically for carers?

3) What is your role as a carer?

4) What impact has being a carer had on your own life?

5) What kind of support do you need as a carer?

6) Have you received any training for the role of carer?

7) Did / do you have any worries or concerns?

**Facilitator Questions** **- Patients**

1) When you were diagnosed with vasculitis, what informational needs did you have?

2) Have you been given any specific information/ leaflets?

3) What is your carer’s role?

4) What impact has having a carer had on your own life?

5) What impact do you think this had had on your carer’s life?

6) Have they received any training for the role of carer?

7) Did / do you have any worries or concerns?
